# GPIHBP1 Autoantibody‐Related Hypertriglyceridemia in a 12‐Year‐Old Girl With Systemic Lupus Erythematosus

**DOI:** 10.1155/crie/6673352

**Published:** 2026-03-02

**Authors:** Sin-ting Tiffany Lai, Suk-yan Suki Chan, Stephanie C. Y. Yu, Jenny Yeuk Ki Cheng, Kam-chi Teresa Tsui, Chi-hang Assunta Ho, Ho-chung Yau

**Affiliations:** ^1^ Department of Paediatrics, Prince of Wales Hospital, The Chinese University of Hong Kong, Hong Kong, China, cuhk.edu.hk; ^2^ Department of Chemical Pathology, Prince of Wales Hospital, The Chinese University of Hong Kong, Hong Kong, China, cuhk.edu.hk

**Keywords:** GPIHBP1 autoantibody, hyperchylomicronemia, hypertriglyceridemia, systemic lupus erythematosus

## Abstract

Glycosylphosphatidylinositol‐anchored high‐density lipoprotein‐binding protein 1 (GPIHBP1) is critical for transporting lipoprotein lipase (LPL) to the capillary lumen, where LPL breaks down triglycerides in triglyceride‐rich lipoproteins. We herein report a 12‐year‐old Chinese girl who presented with severe hypertriglyceridemia and a recent diagnosis of systemic lupus erythematosus (SLE). She was first noted to have severe hypertriglyceridemia at 8.5 years old, complicated by three episodes of acute pancreatitis within 2 years. Between these episodes, her plasma triglycerides remained elevated, but at lower levels. Next‐generation sequencing for primary hypertriglyceridemia yielded no significant findings. Investigations for secondary causes, to include fasting glucose, HbA1c, and thyroid function testing, were unrevealing. Given the fluctuating triglyceride levels and negative genetic testing for primary hypertriglyceridemia in the background of SLE, autoimmune hypertriglyceridemia was suspected. The diagnosis of GPIHBP1 autoantibody syndrome was confirmed by an elevated GPIHBP1 autoantibody titer and a low LPL mass in her serum. Her SLE was well controlled with immunosuppressants and belimumab. Fenofibrate and omega‐3 fatty acids, which were initially prescribed for her hypertriglyceridemia, were later discontinued. The GPIHBP1 autoantibody and LPL mass normalized 2 years after diagnosis. This case illustrates hypertriglyceridemia caused by a rare disease entity associated with autoantibodies against the GPIHBP1 protein. This entity is worth considering after excluding genetic and common secondary causes of hypertriglyceridemia, particularly in a patient with a history of autoimmune disease.

## 1. Introduction

Triglycerides (TGs) serve as a significant energy source for humans, readily hydrolyzed into free fatty acids that generate energy through fatty acid oxidation. TGs are acquired from the diet and synthesized by the liver. Since TGs are highly hydrophobic, they are primarily transported by chylomicrons (exogenous pathway) and very‐low‐density lipoproteins (VLDL; endogenous pathway) [1]. These circulating TG‐rich lipoproteins are hydrolyzed by lipoprotein lipase (LPL) at the endothelial surface of capillaries for subsequent energy use or storage.

Overproduction or impaired clearance of TG‐rich lipoproteins can lead to hypertriglyceridemia. While mild to moderate hypertriglyceridemia is more closely related to premature cardiovascular risk, severe hypertriglyceridemia, defined as fasting plasma TG ≥ 11.3 mmol/L (1000 mg/dL), is associated with an increased risk of pancreatitis, which is potentially fatal [2]. Causes of severe hypertriglyceridemia in pediatric patients can be congenital, acquired, or both. Congenital causes include familial chylomicronemia syndrome (FCS) and transient infantile hypertriglyceridemia [3]. FCS, which is rare, results from biallelic loss‐of‐function variants in genes associated with LPL function (e.g., *LPL*, *LMF1*, *GPIHBP1*, *APOC2*, and *APOA5*). More common is multifactorial chylomicronemia syndrome (MCS), a heterogeneous condition arising from a complex interplay between genetic susceptibility and acquired factors affecting TG metabolism, including obesity and diabetes. Acquired causes secondary to autoantibodies against LPL, glycosylphosphatidylinositol‐anchored high‐density lipoprotein‐binding protein 1 (GPIHBP1), or apolipoprotein (apo) C‐II, collectively termed autoimmune hypertriglyceridemia, have been reported previously [4–13]. The manifestation of autoimmune hypertriglyceridemia may precede or follow the diagnosis of other autoimmune diseases, such as Sjögren’s syndrome, Hashimoto’s thyroiditis, or systemic lupus erythematosus (SLE) [6, 8, 14, 15]. Given its rarity, autoimmune hypertriglyceridemia may be overlooked. We presented a young girl with recurrent acute pancreatitis related to hypertriglyceridemia caused by GPIHBP1 autoantibodies. A diagnosis of SLE was made only after several episodes of acute pancreatitis.

## 2. Case Presentation

A 12‐year‐old Chinese girl with a recent diagnosis of SLE was admitted for management of hypertriglyceridemia.

She was born at 33 weeks of gestation with a birth weight of 2.2 kg (appropriate for gestational age), requiring a hospital stay for respiratory distress and neonatal jaundice. The antenatal history was unremarkable. She was born to non‐consanguineous parents with an unremarkable family history.

**Figure 1:**
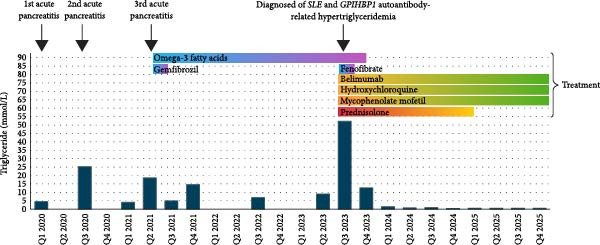
A timeline showing changes in triglyceride levels, episodes of acute pancreatitis, diagnosis of SLE, GPIHBP1 autoantibody‐related hypertriglyceridemia, and treatment. Abbreviation: GPIHBP1, glycosylphosphatidylinositol‐anchored high‐density lipoprotein‐binding protein 1; SLE, systemic lupus erythematosus.

She was in good health until 4 years ago. She was admitted for three episodes of hypertriglyceridemia‐related acute pancreatitis at ages 8.5, 9.5, and 10 treated conservatively in outside hospitals with uneventful recoveries. During these acute episodes, her TG levels were significantly elevated, reaching as high as 25.4 mmol/L (2247.8 mg/dL). The total cholesterol (TC) and high‐density lipoprotein‐cholesterol (HDL‐C) levels were within normal limits. Upon discharge from these acute episodes, her TG remained elevated but at lower levels, ranging from 4.1 to 5.6 mmol/L (362.8 to 495.6 mg/dL) (Figure 1).

After her first episode of acute pancreatitis, she was noted to have an elevated anti‐nuclear antibody (ANA) (1:640) and anti‐dsDNA (3+). Multiple next‐generation sequencing analyses could only identify a variant of uncertain significance in the APOB gene (NM_000384.3(APOB):c.107G > C p.(Ser36Thr)), which was not accountable for her phenotype. The patient was advised to follow a low‐fat diet since her first admission. After her third admission, she was started on gemfibrozil, which was later discontinued due to elevated alanine aminotransferase and aspartate aminotransferase levels. She then started taking omega‐3 fatty acids and traditional Chinese medicine. She remained asymptomatic, with TG levels ranging from 7 to 9 mmol/L (619.5 to 796.5 mg/dL).

1 month prior to her presentation to our unit, she visited an outside hospital for a fever and malar rash. Investigations revealed pancytopenia and positive lupus serologies, including an elevated ANA titer, positive anti‐dsDNA and anti‐smooth muscle antibodies, a positive direct Coombs test, and low levels of C3 and C4 complement. She was diagnosed with SLE and was treated with methylprednisolone followed by prednisolone, hydroxychloroquine, mycophenolate mofetil, and belimumab. She was also found to have hypertriglyceridemia. Her TG level upon admission was 4.6 mmol/L (407.1 mg/dL), which surged to 47.7 mmol/L (4221.2 mg/dL) 10 days later and peaked at 52.4 mmol/L (4637.2 mg/dL) on day 12. She underwent plasmapheresis twice for uncontrolled SLE and severe hypertriglyceridemia, decreasing her TG level to 12.9 mmol/L (1141.6 mg/dL) on day 14 of her stay. While SLE control was satisfactory, her TG level rebounded to 42.6 mmol/L (3769.9 mg/dL) on day 24. She was discharged against medical advice from the outside hospital and subsequently admitted to our unit for further management. Medications before discharge included oral prednisolone (30 mg/day), mycophenolate mofetil (1000 mg/day), and hydroxychloroquine (150 mg/day).

Upon admission to our unit, she was asymptomatic. Her BMI was 18.5 kg/m^2^. Physical examination revealed a fading malar rash with no clinical signs of hyperlipidemia. Her complete blood count was normal. The fasting lipid profile showed a TC level of 5.0 mmol/L (193.1 mg/dL), a TG level of 10.5 mmol/L (929.2 mg/dL), an HDL‐C level of 0.5 mmol/L (19.3 mg/dL), a direct LDL‐C level of 1.0 mmol/L (38.6 mg/dL; in the context of hypertriglyceridemia, both direct and calculated LDL‐C levels may not accurately reflect the actual LDL‐C level), an Apo A1 of 0.83 g/L (normal: 1.2 – 2.0 g/L), and an Apo B of 0.77 g/L (normal: 0.41 – 1.07 g/L). SLE treatment was optimized to include oral prednisolone 25 mg/day, mycophenolate mofetil 1500 mg/day, hydroxychloroquine 150 mg/day, and intravenous belimumab 10 mg/kg every 4 weeks. She continued on a low‐fat diet and started on oral fenofibrate 145 mg/day and omega‐3 fatty acids 2 g/day. Upon discharge, her TG was 6.1 mmol/L (539.8 mg/dL).

Lipoprotein electrophoresis of the fasting plasma sample obtained during admission to our unit revealed a chylomicron band and a VLDL band. Investigations for secondary causes of hypertriglyceridemia, including thyroid function testing, fasting blood glucose, HbA1c, and liver and renal function tests, were unremarkable. Given the fluctuating plasma TG levels, the negative workup for more common causes of secondary hypertriglyceridemia, the negative genetic workup for primary hypertriglyceridemia, and a recent diagnosis of SLE, suspicion for autoimmune hypertriglyceridemia, potentially due to autoantibodies against LPL, apo C‐II, or GPIHBP1, was raised. Plasma samples obtained during severe hypertriglyceridemia (TG 12.6 and 11.6 mmol/L [1115 and 1026.6 mg/dL, respectively]) were retrieved for testing. The findings of elevated GPIHBP1 autoantibody titers (1206.0 and 846.7 U/mL, respectively; normal: <58.4 U/mL) (ELISA; Immuno‐Biological Laboratories, Fujioka, Japan) and low LPL mass (8.0 and 6.4 ng/mL, respectively; normal: 26.5 – 105.5 ng/mL)(ELISA; Immuno‐Biological Laboratories, Fujioka, Japan) confirmed the diagnosis of hypertriglyceridemia secondary to GPIHBP1 autoantibodies (Table 1) [6, 16].

**Table 1:** Lipid profile, GPIHBP1 antibody, GPIHBP1 mass, LPL mass, ANA titer, anti‐dsDNA titer, anti‐ENA, and anti‐Ro from diagnosis to 2‐year post‐treatment.

Test/treatment	Unit	At diagnosis	On discharge	1m	2m	3m	4m	6m	9m	12m	15m	18m	21m	24m
Lipid profile														
Triglyceride	(mmol/L)	12.6	6.1	1.2	1.8	0.9	0.7	1.1	0.9	0.6	0.7	0.6	0.7	0.7
	(mg/dL)	1115	539.8	106.2	159.3	79.7	62	97.4	79.7	53.1	62	53.1	62	62
Total cholesterol	(mmol/L)	5.4	3.4	3.8	4.3	4.9	4.9	4.8	4.1	3.9	4.5	4.3	4.6	4.1
	(mg/dL)	208.5	131.3	146.7	166	189.2	189.2	185.3	158.3	150.6	174	166.3	177.9	158.3
HDL‐Cholesterol	(mmol/L)	0.5	0.5	1.3	1.6	1.5	1.5	1.6	1.6	1.4	1.7	1.7	1.6	1.6
	(mg/dL)	19.3	19.3	50.2	61.8	57.9	57.9	61.8	61.8	54.1	65.7	65.7	61.8	61.8
LDL‐cholesterol	(mmol/L)	1.1^a^		2.0	1.9	3.0	3.0	2.7	2.1	2.2	2.5	2.4	2.7	2.2
	(mg/dL)	42.5^a^		77.2	73.4	115.8	115.8	104.3	81.1	84.9	96.7	92.8	104.4	84.9
Non‐HDL‐cholesterol	(mmol/L)	4.9		2.5	2.7	3.3	3.3	3.2	2.5	2.5	2.8	2.6	3.0	2.5
	(mg/dL)	189.2		96.5	104.3	127.4	127.4	123.6	96.5	96.5	108.1	100.4	115.8	96.5
GPIHBP1 antibody (<58.4)	(U/ml)	1206												2.6
GPIHBP1 mass (570.6–1625.6)	(pg/ml)	98.5												721.1
LPL mass (26.5–105.5)	(ng/ml)	8.0												52.4
ANA titer	(IU/ml)	320		160		160	160	80	80	40	40	40	80	40
Anti‐ds‐DNA titer (<100)	(IU/ml)	351		66		68	47	32	21	12	13	<10	<10	<10
Anti‐ENA		Positive				Positive		Negative	Negative	Negative	Negative	Negative	Negative	Positive
Anti‐Ro		Positive				Weakly positive		Negative	Negative	Negative	Negative	Negative	Negative	Negative
SLE treatment														
Prednisolone	(mg/day)	25	25	15	10	7.5	7.5	5	3	2.5	2.5	—	—	—
Mycophenolate mofetil	(mg/day)	1500	1500	1500	1500	1500	1500	1500	1500	1500	1500	1250	1250	1250
Hydroxychloroquine	(mg/day)	150	150	150	150	200	200	200	200	200	200	200	200	200
Belimumab	(mg/kg/4 weeks)	10	10	10	10	10	10	10	10	10	10	10	10	10
Lipid‐lowering agents														
Omega‐3 fatty acids	(g/day)	2	2	2	2	1	1	—	—	—	—	—	—	—
Fenofibrate	(mg/day)	145	145	72.5	72.5	—	—	—	—	—	—	—	—	—

Abbreviations: ANA, anti‐nuclear antibody; Anti‐ENA, anti‐extractable nuclear antibody; GPIHBP1, glycosylphosphatidylinositol‐anchored high‐density lipoprotein‐binding protein 1; LPL, lipoprotein lipase; m, month.

^a^Direct LDL‐C.

Her SLE and hypertriglyceridemia were well controlled with immunosuppressants and belimumab. 4 months into treatment, her plasma TG levels normalized, and she was weaned off fenofibrate and omega‐3 fatty acids. At 1.5 years after diagnosis, her ANA titer decreased from 320 to 40 IU/mL, and her anti‐dsDNA titer became undetectable. Her prednisolone was tapered off, mycophenolate mofetil was continued at 1250 mg/day, hydroxychloroquine at 200 mg/day, and belimumab at 10 mg/kg every 4 weeks. While on the same treatment regimen, her GPIHBP1 autoantibody titer (2.6 U/mL; normal: <58.4 U/mL) and LPL mass (52.4 ng/mL; normal: 26.5 – 105.5 ng/mL) normalized at 2 years after diagnosis (Table 1, Figure 1).

## 3. Discussion

GPIHBP1 is a glycoprotein expressed on the surface of capillary endothelial cells. It binds to LPL in the interstitial space and shuttles it to the capillary lumen for TG hydrolysis [17]. Autoantibodies against GPIHBP1 inhibit LPL binding. As a result, no functional LPL is available in the capillary lumen. This prevents the breakdown of TG in chylomicrons and VLDL, leading to severe hypertriglyceridemia [6]. Hypertriglyceridemia caused by autoantibodies against GPIHBP1 was first discovered in 2017 [6]. Currently, around 30 cases have been published (Table 2) [6–11, 14, 15, 18–21]. Due to the impaired delivery of LPL to the capillary lumen, patients with GPIHBP1 autoantibodies have low plasma LPL levels [6, 14]. The diagnosis of GPIHBP1 autoantibody syndrome is confirmed by the detection of GPIHBP1 autoantibodies and supported by a low LPL mass [16]. Autoimmune hypertriglyceridemia resulting from autoantibodies against LPL or apoC‐II is unlikely, as LPL mass is typically normal in these conditions [12]. Patients with GPIHBP1 autoantibody syndrome experience intermittent episodes of hypertriglyceridemia, and the disappearance of the autoantibody coincides with the normalization of TG and LPL mass [20, 21]. Nonetheless, the GPIHBP1 autoantibody titer does not appear to correlate with the severity of hypertriglyceridemia [14].

**Table 2:** Literature review on patients diagnosed with GPIHBP1 autoantibody‐related hypertriglyceridemia.

Patient	Ref.	Sex	Age^a^	Autoimmune disease	Hypertriglyceridemia and acute pancreatitis	Immunosuppressant treatment	Follow‐up GPIHBP1 antibody	Follow‐up TG (mmol/L) [mg/dl]
1 [patient 101]	[6]	F	53	Rheumatoid arthritis and Sjogren’s syndrome diagnosed at age 34; Hashimoto thyroiditis and SLE diagnosed at age 40	Hypertriglyceridemia and acute pancreatitis presented at age 34 and 40 respectively	Prednisolone (10 mg/day), Salazosulfapyridine (1000 mg/day)	—	8.2–56.3 (723–4980)
2 [patient 111]	[6]	F	20	Nil	Hypertriglyceridemia and recurrent pancreatitis presented at age 5; resistant to fenofibrate and omega‐3 fatty acid supplement	Mycophenolate mofetil	—	29.1–46.2 (2573–4085)
3 [patient 164]	[6]	F	—^b^	Diagnosed with Sjogren’s syndrome 18 months after the presentation of hypertriglyceridemia	Hypertriglyceridemia and acute pancreatitis presented at age 9; resistant to a low‐fat diet, fenofibrate (145 mg daily) and omega‐3 fatty acid supplement (2 g/day)	Prednisolone (20 mg daily, later tailed off), Mycophenolate mofetil (1000 mg/day, increased to 1250 mg/day), Hydroxychloroquine (100 mg/day)	—	0.4 (37)
4 [patient 102]	[6]	F	38	Diagnosed with SLE 2 years before the presentation of hypertriglyceridemia	Hypertriglyceridemia and acute pancreatitis at 33 weeks of pregnancy; required plasmapheresis	Prednisone (5 mg/day)	—	—
5 [patient 103]	[6]	F	0	Neonatal lupus (maternal GPIHBP1 autoantibodies causing chylomicronemia)	Baby of patient 4, hypertriglyceridemia placed on medium chain triglyceride‐rich formula	No	—	0.8 (72)
6 [patient 157]	[6]	F	—^c^	Diagnosed with SLE (at 12 years old) before the presentation of hypertriglyceridemia	Hypertriglyceridemia and recurrent pancreatitis presented at age 26; required plasmapheresis	Prednisolone, Azathioprine, cyclophosphamide, Rituximab (375 mg/m weekly for 4 doses)	—	—
7 [patient 38]	[6]	M	—^c^	Nil	Hypertriglyceridemia and recurrent pancreatitis presented at age 16, resistant to a low‐fat diet, fibrate therapy and omega‐3 fatty acid supplement	No	—	—
8	[7]	M	36	Nil	Hypertriglyceridemia and acute pancreatitis diagnosed at around age 30	No	—	—
9	[8]	F	18	Diagnosed with SLE around 3 years after the presentation of hypertriglyceridemia	Hypertriglyceridemia and acute pancreatitis presented at age 15, resistant to a low‐fat diet, fenofibrate, ursodeoxycholic acid, and omega‐3 fatty acid supplement	Prednisolone (10 mg/day)	—	—
10	[9]	F	35	Diagnosed with autoimmune thyroiditis before the presentation of hypertriglyceridemia	Hypertriglyceridemia presented at age 35, treated with a low‐fat diet, fenofibrate 80 mg/day and omega‐3 fatty acid supplement (4 g/day)	No	—	9 (800)
11	[10]	M	44	Diagnosed with Immune thrombocytopenia purpura (at age 41) before the presentation of hypertriglyceridemia	Hypertriglyceridemia presented at age 44 after discontinuation of prednisolone	Prednisolone (15 mg/day)	—	2.3 (200)
12	[11]	F	27	Diagnosed with Hashimoto thyroiditis (at age 26) before the presentation of hypertriglyceridemia	Hypertriglyceridemia and acute pancreatitis presented at age 27, resistant to pemafibrate (0.2–0.4 mg/day) and eicosapentaenoic acid (1800 mg/day)	Rituximab (375 mg/m^2^ at 1 to 2 weeks intervals for 4 doses)	Undetectable^d^	2.3 (200)
13 [ID no. 8]	[14]	F	3	Hemolytic anemia	—	—	—	—
14 [ID no. 9]	[14]	F	23	ANA+	—	—	—	—
15 [ID no. 10]	[14]	M	50	Hemolytic anemia	—	—	—	—
16 [ID no. 12]	[14]	F	11	Nil	Acute pancreatitis	—	—	—
17 [ID no. 14]	[14]	F	28	ANA+	—	—	—	—
18 [ID no. 15]	[14]	F	15	SLE, Anti phospholipid syndrome	—	Rituximab	Positive response	—
19 [ID no. 16]	[14]	M	11	Hashimoto thyroiditis	—	—	—	—
20 [ID no. 17]	[14]	F	15	ANA+	Acute pancreatitis	—	—	—
21 [ID no. 18]	[14]	F	35	Hashimoto thyroiditis	—	—	—	—
22 [ID no. 19]	[14]	F	29	Hashimoto thyroiditis	—	—	—	—
23 [ID no. 20]	[14]	F	14	SLE	Acute pancreatitis	—	—	—
24 [ID no. 21]	[14]	M	13	ANA+	—	—	—	—
25 [ID no. 22]	[14]	F	14	Suspected SLE/ rheumatoid arthritis	Acute pancreatitis	Rituximab	Positive response	—
26	[15]	F	14	Diagnosed with SLE (at around age 15) after the presentation of hypertriglyceridemia	Hypertriglyceridemia and recurrent pancreatitis diagnosed at around age 14, initiated on a low‐fat diet, omega‐3 fatty acid and MCT oil supplement, later added on fenofibrate (up to 160 mg/day)	Prednisolone (50 mg/day, tailed down to 25 mg/day), Mycophenolate mofetil (500 mg/day) increased to 1500 mg/day)	Normalized	1.1 (97)
27	[18]	F	34	Multiple sclerosis	Hypertriglyceridemia during IFNß1a therapy for multiple sclerosis	No	Undetectable^e^	Normalized^e^
28	[20]	F	27	Antiphospholipid syndrome, Garve’s disease before the presentation of hypertriglyceridemia	Hypertriglyceridemia (>5500 mg/dL), resistant to a low‐fat diet, fenofibrate (250 mg/day), ezetimibe (10 mg/day) and plasmapheresis	Rituximab (375 mg weekly for 2 dose, with 3^rd^ dose 6 months after)	Undetectable	Normalized
29	[21]	F	16	Nil	Hypertriglyceridemia and recurrent pancreatitis diagnosed at age 15, treated with a low‐fat diet and fenofibrate	Mycophenolate mofetil (2 g/day) Prednisolone (60 mg/day taper to 40 mg/day) Rituximab (1 g) and Solu‐Medrol (1 g) for 2 dose	Undetectable	1.2 (105)

Abbreviations: ANA, anti‐nuclear antibody; GPIHBP1, glycosylphosphatidylinositol‐anchored high‐density lipoprotein‐binding protein 1; MCT, medium‐chain triglyceride; No., number; Ref., reference; SLE, systemic lupus erythematosus; TG, triglyceride.

^a^Age at diagnosis of GPIHBP1 autoantibody‐related hypertriglyceridemia.

^b^Initially reported as Anti‐LPL‐related hypertriglyceridemia.

^c^Deceased before the diagnosis of GPIHBP1 autoantibody‐related hypertriglyceridemia.

^d^Before the initiation of immunosuppressant.

^e^After termination of IFNß1a therapy.

Although sometimes reported as an isolated condition [6, 7, 14, 21], patients often have pre‐existing autoimmune disorders, such as rheumatoid arthritis, SLE, Sjögren’s syndrome, antiphospholipid syndrome, Hashimoto’s thyroiditis, Graves’ disease, Basedow’s syndrome, and multiple sclerosis following interferon treatment [6–11, 14, 15, 18, 20, 21]. Our patient experienced recurrent hypertriglyceridemia‐related acute pancreatitis since the age of 8.5 years. She was ultimately diagnosed with SLE and GPIHBP1 autoantibody‐related hypertriglyceridemia at age 12 (Figure 1). We assert that the previous episodes of severe hypertriglyceridemia complicated by acute pancreatitis were also attributable to GPIHBP1 autoantibodies, which occurred 3.5 years before the diagnosis of SLE. A similar observation, where the presentation of hypertriglyceridemia flares preceded the diagnosis of an autoimmune disorder, has been previously reported (Table 2) [6, 8, 15]. It remains unclear whether the disease activity of the associated autoimmune disorder correlates with the hypertriglyceridemia flares. Monitoring SLE disease markers, serum TG levels, and GPIHBP1 autoantibody titers over time in our patient is crucial for determining whether SLE disease activity is associated with flares of hypertriglyceridemia.

Our patient was started on belimumab for her treatment of SLE, a monoclonal antibody against B‐lymphocyte stimulator that depletes B cells. Her TG level nearly normalized 1 month after starting treatment, around which the diagnosis of GPIHBP1 autoantibody‐related hypertriglyceridemia was confirmed. We thus continued belimumab, and her serum TG level remained normal 2 years later. In addition, her anti‐dsDNA titer, as well as her GPIHBP1 autoantibody titer and LPL mass, were normalized at 1.5 years and 2 years after diagnosis, respectively (Table 1). To date, although reports have been published on the normalization of TG and the disappearance of autoantibodies using rituximab (Table 2), the optimal management of GPIHBP1 autoantibody‐related hypertriglyceridemia remains controversial [7, 11, 14, 20, 21]. There were also reports of normalization of serum TG levels and/or disappearance of GPIHBP1 autoantibody without [6, 7] or before [11] the use of immunosuppressants (Table 2). Our case demonstrated that belimumab, a biologic treatment for SLE, is also effective in treating GPIHBP1 autoantibody‐related hypertriglyceridemia and could be considered for patients with both conditions.

Corticosteroids, commonly used to treat autoimmune disorders such as SLE, may worsen hypertriglyceridemia by increasing insulin resistance, which leads to increased VLDL synthesis in the liver and reduced VLDL breakdown due to decreased LPL activity. However, in our patient, corticosteroids suppress SLE disease activity and help control serum TG levels.

In conclusion, detection of the GPIHBP1 autoantibody is essential for diagnosing GPIHBP1 autoantibody‐related hypertriglyceridemia and is supported by low LPL levels. Autoimmune hypertriglyceridemia should be considered as a differential diagnosis in patients experiencing acute or intermittent episodes of hypertriglyceridemia when genetic and secondary causes are excluded, especially in those with pre‐existing autoimmune conditions or serological markers such as ANA positivity. Conversely, hypertriglyceridemia could be the first sign of an evolving autoimmune disease. Close monitoring of patients with isolated hypertriglyceridemia caused by autoantibodies against GPIHBP1 may help facilitate early detection of an underlying autoimmune disorder.

**Nomenclature**
Apo: Apolipoprotein
FCS: Familial chylomicronemia syndrome
GPIHBP1: Glycosylphosphatidylinositol‐anchored high‐density lipoprotein‐binding protein 1
HDL‐C: High‐density lipoprotein‐cholesterol
LDL‐C: Low‐density lipoprotein‐cholesterol
LPL: Lipoprotein lipase
SLE: Systemic lupus erythematosus
TC: Total cholesterol
TG: Triglyceride
VLDL: Very‐low‐density lipoprotein
WES: Whole exome equencing.

## Consent

Signed informed written consent for publication of clinical details and/or clinical images was obtained directly from the patient and the patient’s guardian.

## Disclosure

The authors have nothing to report.

## Conflicts of Interest

The authors declare no conflicts of interest.

## Author Contributions

Conception: Sin‐ting Tiffany Lai, Ho‐chung Yau, Kam‐chi Teresa Tsui. Data collection: Sin‐ting Tiffany Lai, Suk‐yan Suki Chan, Chi‐hang Assunta Ho, Kam‐chi Teresa Tsui, Ho‐chung Yau. Data Presentation: Sin‐ting Tiffany Lai. Administrative support: Kam‐chi Teresa Tsui. Manuscript writing: Sin‐ting Tiffany Lai, Stephanie C. Y. Yu, Jenny Yeuk Ki Cheng. Manuscript editing: Sin‐ting Tiffany Lai, Suk‐yan Suki Chan, Stephanie C. Y. Yu, Jenny Yeuk Ki Cheng, Kam‐chi Teresa Tsui, Chi‐hang Assunta Ho, Ho‐chung Yau. Final approval: Sin‐ting Tiffany Lai, Suk‐yan Suki Chan, Stephanie C. Y. Yu, Jenny Yeuk Ki Cheng, Kam‐chi Teresa Tsui, Chi‐hang Assunta Ho, Ho‐chung Yau.

## Funding

The research and preparation of this manuscript did not receive any specific grant from funding agencies in the public, commercial, or not‐for‐profit sectors.

## Data Availability

Data sharing does not apply to this article, as no new data were created or analyzed in this study.
